# Link between perceived oral and general health status among Yemeni adult dental patients

**DOI:** 10.1186/s12903-019-0793-6

**Published:** 2019-05-28

**Authors:** Mohammed Nasser Alhajj, Esam Halboub, Abdullah G. Amran, Abdulaziz A. Alkheraif, Fuad A. Al-Sanabani, Bandar M. Al-Makramani, Abdulghani A. Al-Basmi, Fawaz A. Al-Ghabri

**Affiliations:** 1 Department of Prosthodontics, Faculty of Dentistry, Thamar University, Dhamar, Yemen; 2 Department of Maxillofacial Surgery and Diagnostic Sciences, College of Dentistry, Jazan University, Jazan, Kingdom of Saudi Arabia; 3 Department of Periodontics, Faculty of Dentistry, Thamar University, Dhamar, Yemen; 4 Dental Biomaterials Research Chair, Dental Health Department, College of Applied Medical Sciences, King Saud University, Riyadh, Kingdom of Saudi Arabia; 5 Department of Prosthetic Dental Science, College of Dentistry, Jazan university, Jazan, Kingdom of Saudi Arabia; 6 Private Dental Clinic, Dhamar, Yemen

## Abstract

**Background:**

Self-perceived health is an essential measure of health status and even a paramount predictor of mortality. So long as it is said that oral health (OH) and general health (GH) are mirrors to each other. This study sought to determine how Yemeni adults rate their OH and GH, whether such a self-rating influenced by some potential risk factors, and whether both ratings (OH and GH) are correlated.

**Methods:**

A sample of 587 Yemeni dental patients aged 20 years and over were consecutively recruited. A structured interview form was used covering the following variables: age, gender, marital status, educational level, presence of dental prosthesis (DP), smoking and Qat chewing habits as independent variables, along with questions on “perceived oral health (POH)” and “perceived general health (PGH)” as dependent variables. The bivariate and multiple ordinal regression analyses were applied at *P*-value < 0.05.

**Results:**

Most of participants were women (73.6%), and married (71.4%), and more than half of them were young adults (58.2%), with high educational levels (53.3%), and not having DP. Only 310 participants responded to the questions on smoking and Qat chewing habits. Of these, 88.5% were non-smokers and 62.1% were Qat non-chewers. Up to 50% of the participants reported their POH as poor or fair, while lower proportions of participants (17%) reported their PGH as such. Younger age (compared to elders), high education levels (compared to primary education) and being single (compared to married) significantly revealed better levels of POH, while high education levels and being females significantly revealed better levels of PGH. Smoking and Qat chewing habits were found to have no effect on the perception of POH or PGH. POH and PGH were found to be significantly correlated (*r* = 0.486; *P* < 0.001).

**Conclusion:**

Higher levels of oral health problems can be anticipated among patients who perceive poor general health, and vice versa. The age, marital status and education were independent determinants of POH, while the gender and education were independent determinants of PGH.

## Background

Based on World Health Organization (WHO), health is defined as “a state of complete physical, mental and social well-being and not merely the absence of disease or infirmity” [1, 2]. Although different definitions have also been suggested [3–6], they are still very broad, complex, and multidimensional [4, 5, 7, 8]; no consensus on a single definition has been reached. Huber et al. [9] provided acceptable and concise definition for health as “the ability to adapt and to self-manage.” The above argument is about health in general (General Health [GH]) of which oral health (OH) is an essential part. However, WHO [10] defined the latter as “a state of being free from mouth and facial pain, oral and throat cancer, oral infection and sores, periodontal (gum) disease, tooth decay, tooth loss, and other diseases and disorders that limit an individual’s capacity in biting, chewing, smiling, speaking, and psychosocial wellbeing.” For its part, the American dental association defines OH as “a functional, structural, aesthetic, physiologic and psychosocial state of well-being and is essential to an individual’s general health and quality of life” [11].

As a rule, GH and OH cannot be viewed separately. Indeed, they are integral of each other; OH is considered as a mirror through which the GH is reflected in most instances. In fact, several systemic diseases have oral manifestations - might be as the early signs -, and many oral diseases have different effects on GH. For instance, many oral/periodontal pathogens have been reported to predispose to cardiovascular diseases [12, 13], and respiratory diseases such as chronic bronchitis, pneumonia, and chronic pulmonary obstructive disease (CPOD) [14, 15]. In line with that, diabetic patients may develop severe inflammation of the gum and periodontal tissues, and presence of periodontal infection in these patients may exacerbate the case and complicate blood glucose control [16, 17]. Although not particular to AIDS, oral candidiasis and necrotizing ulcerative periodontitis are diagnosed more frequently among these patients. [18]. Similarly, dysfunction of temporomandibular joint (TMJ) may be a part of a more generalized involvement - osteoarthritis for example -, and bone mass reduction in the mandible has been detected as a sign of osteoporosis [19, 20].

It can be noticed that all definitions of GH or OH are more than being free of diseases. They relates to many aspects in the individual’s daily life. Presence of diseases, medications, functional ability, obesity, psychological wellbeing, and socioeconomic factors (alcohol consumption, smoking, etc.) can alter the individual’s health status [21–23]. Health can be either subjectively perceived (self-rated) or assessed objectively by a physician using specific tools or indicators. Self-rated or perceived health is the summary of all information available to individual about his/her current health status [24, 25]. Perceived health can be measured by a single question: a) age-comparative question where subjects are asked to compare their health with someone else of the same age; b) time-comparative question where subjects are asked to compare their current health with that in the past; and c) non-comparative question where subjects are asked to rate their health at that moment [26, 27]. The non-comparative question is the most commonly used in research and clinical practice. It is a simple question directed to the participants about their feelings about oral/general health status.

In this context, several studies have been conducted among different populations to address this issue with variable results. A study of Kotha et al. (2017) in Saudi Arabia revealed 75.1% of the participants rated their OH as excellent/very good/good; 24.9% rated their OH as poor to fair [28]. In South Africa, Olutola and AyoYusuf reported a proportion of76.3% of their study sample rated their OH as good [29], almostly similar to what Andrade et al. (2012) reported among Brazilian sample (74.36%) [30]. Up to 83% of Australian adults sample who questioned by Mejia et al. (2014) rated their OH as good while it was reported only by 49% of an Indian sample [31, 32] and by 58.3% of a Nigerian sample [33]. Assessment of patients’ perception about their oral and general health is of utmost importance. It gives clear idea about the patients’ feeling about their health. This can be reflected on the objective examination and ensure ideal or at least acceptable treatment from the patients’ point of view. The aims of this study, therefore, were to determine how Yemeni adults rate their OH and GH, and whether such a self-rating influenced by some potential risk factors.

## Methods

The study was of a cross-sectional design in which the study population were dental patients attendees of outpatient clinics, Faculty of Dentistry, Thamar University, and three private clinics in Thamar city. During the period from June to September 2018, a consecutive sample comprised 587 participants were recruited. Eligibility criteria were: being 18 years old and above, Yemeni nationality, with no mental or physical disability. They were explicitly informed about the study objectives and procedure, and that the confidentiality of their data was guaranteed. Those who agreed to take a part in this study signed informed consents accordingly. The study was approved by the Ethical Committee, Thamar University, ahead of commencing its procedures (Ref: 2018019).

A structured interview form was used covering the following variables: age, gender, marital status, educational level, presence of dental prosthesis, smoking and Qat chewing habits as independent variables, along with the global single questions of “perceived oral health (POH)” (How do you rate your oral health status?) and “perceived general health (PGH)” (How do you rate your general health status?) as dependent variables. Responses to POH and PGH were scored as: 5 = excellent, 4 = very good, 3 = good, 2 = fair, and 1 = poor. The age was categorized as: ≤ 20, 21–30, 31–40, 41–50, and >  50 years. The educational status included illiterate, primary, preparatory, secondary, and university or above. The following dental prostheses (DP) were asked about: fixed DP, removable DP, implants, or more than one DP; responses to DP question were coded as: “yes” or “no”. And similarly were coded the responses to smoking and Qat chewing.

### Statistical analysis

Data were gathered, coded, and entered to a master sheet (Excel 2013, Microsoft). The statistical software SPSS V.25 (IBM Corp, Armonk, NY) was used for data analysis. For descriptive analysis, data were presented as frequency and percentage. Non-parametric tests were used for inferential analysis. Chi-squared test was used to test the potential association between POH and PGH, and between any of them (dependent variables) with the different study variables (independent variables). For the former test, contingency coefficient was checked and linear by linear results were selected. Additionally, Spearmen’s correlation coefficient test was used to test the potential correlation between POH and PGH. Ordinal regression analysis with adjusted odds ratio (OR) was performed to explore the dependent determinants and the effect of the study variables on POH and PGH. All statistical tests were performed at a significant level *P*-value < 0.05.

## Results

Table 1 presents the characteristics of the participants. Out of 587, 73.6% were women, 58.2% were young adults (≤30 years), and 71.4% were married. With regard to the education, secondary and university or above levels were reported more frequently (25.9 and 27.4%, respectively). Up to 63% of participants reported not having PD. Only 310 participants responded to the questions on smoking and Qat chewing habits. Of these, 88.5% were non-smokers and 62.1% were Qat non-chewers. Up to 50% of the participants reported their POH as poor or fair, while 30% reported good POH. In contrast, up to 93% of the participants reported their PGH as good, very good or excellent.

**Table 1:** Characteristics of the study sample (*N* = 587 unless otherwise stated)

Age	≤ 20 years	70 (11.9)
	21–30 years	272 (46.3)
	31–40 years	137 (23.3)
	41–50 years	55 (9.4)
	> 50 years	53 (9.0)
Gender	Male	155 (26.4)
	Female	432 (73.6)
Marital status	Single	168 (28.6)
	Married	419 (71.4)
Education	Illiterate	95 (16.2)
	Primary	91 (15.5)
	Preparatory	88 (15.0)
	Secondary	152 (25.9)
	University or above	161 (27.4)
Dental prosthesis	Yes	216 (36.8)
	No	371 (63.2)
Smoking (*N* = 310)	Yes	38 (11.5)
	No	292 (88.5)
Qat chewing (N = 310)	Yes	125 (37.9)
	No	205 (62.1)
Perceived oral health	Poor	138 (23.5)
	Fair	150 (25.6)
	Good	175 (29.8)
	Very good	93 (15.8)
	Excellent	31 (5.3)
Perceived general health	Poor	29 (4.9)
	Fair	69 (11.8)
	Good	205 (34.9)
	Very good	177 (30.2)
	Excellent	107 (18.2)

Higher levels of POH (very good and excellent) were reported more frequently, with statistical significance, by young participants, males, singles, those who had higher levels of education and those who never wear dental prosthesis (*P* < 0.001 each). Also to less extent, higher levels of POH (very good and excellent) were reported by nonsmokers, but it wasn’t statistically significant (*P* = 0.051), and by Qat non-chewers with significant difference compared to Qat chewers (*P* = 0.042; Table 2).

**Table 2:** The distribution of perceived oral health (POH) according to the different variables (*N* = 587 unless otherwise stated)

		Perceived oral health					*P*
		Poor	Fair	Good	Very good	Excellent	
Age	≤ 20 years	10 (14.3)	16 (22.9)	27 (38.6)	12 (17.1)	5 (7.1)	<.001
	21–30 years	61 (22.4)	53 (19.5)	85 (31.3)	53 (19.5)	20 (7.4)	
	31–40 years	34 (24.8)	37 (27.0)	39 (28.5)	22 (16.1)	5 (3.6)	
	41–50 years	12 (21.8)	19 (34.5)	19 (34.5)	4 (7.3)	1 (1.8)	
	> 50 years	21 (39.6)	25 (47.2)	5 (9.4)	2 (3.8)	0 (0.0)	
Gender	Male	18 (11.6)	46 (29.7)	53 (34.2)	22 (14.2)	16 (10.3)	<.001
	Female	120 (27.8)	104 (24.1)	122 (28.2)	71 (16.4)	15 (3.5)	
Marital status	Single	22 (13.1)	35 (20.8)	56 (33.3)	42 (25.0)	13 (7.7)	<.001
	Married	116 (27.7)	115 (27.4)	119 (28.4)	51 (12.2)	18 (4.3)	
Education	Illiterate	48 (50.5)	31 (32.6)	13 (13.7)	3 (3.2)	0 (0.0)	<.001
	Primary	28 (30.8)	33 (36.3)	21 (23.1)	7 (7.7)	2 (2.2)	
	Preparatory	26 (29.5)	21 (23.9)	31 (35.2)	8 (9.1)	2 (2.3)	
	Secondary	22 (14.5)	27 (17.8)	51 (33.6)	35 (23.0)	17 (11.2)	
	University or above	14 (8.7)	38 (23.6)	59 (36.6)	40 (24.8)	10 (6.2)	
Dental prosthesis	Yes	64 (29.6)	67 (31.0)	67 (31.0)	17 (7.9)	1 (0.5)	<.001
	No	74 (19.9)	83 (22.4)	108 (29.1)	76 (20.5)	30 (8.1)	
Smoking (N = 310)	Yes	7 (18.4)	19 (50.0)	9 (23.7)	1 (2.6)	2 (5.3)	0.051
	No	54 (18.5)	74 (25.3)	103 (35.3)	48 (16.4)	13 (4.5)	
Qat chewing (N = 310)	Yes	27 (21.6)	43 (34.4)	36 (28.8)	12 (9.6)	7 (5.6)	0.042
	No	34 (16.6)	50 (24.4)	76 (37.1)	37 (18.0)	8 (3.9)	

Chi-square test was used; POH: perceived oral health

Responses of the participants to PGH question were almost similar to their responses to POH question. Overall, higher levels of PGH (good, very good and excellent) were reported more frequently by young participants (*P* < 0.001), males (*P* = 0.002), singles (*P* < 0.001), those who had higher levels of education (P < 0.001) and those who never wear DP (*P* = 0.001). However, the associations of these responses with smoking and Qat chewing habits were not statistically significant (*P* = 0.904 and 0.250, respectively; Table 3). Single men, however, were found to report their oral and general health status better than single women (*P* < 0.05).

**Table 3:** The distribution of perceived general health (PGH) according to the different variables (*N* = 587 unless otherwise stated)

		Perceived general health					*P*
		Poor	Fair	Good	Very good	Excellent	
Age	≤ 20 years	5 (7.1)	7 (10.0)	14 (20.0)	27 (38.6)	17 (24.3)	<.001
	21–30 years	11 (4.0)	26 (9.6)	86 (31.6)	85 (31.3)	64 (23.5)	
	31–40 years	7 (5.1)	13 (9.5)	58 (42.3)	38 (27.7)	21 (15.3)	
	41–50 years	2 (3.6)	13 (23.6)	20 (36.4)	17 (30.9)	3 (5.5)	
	> 50 years	4 (7.5)	10 (18.9)	27 (50.9)	10 (18.9)	2 (3.8)	
Gender	Male	5 (3.2)	16 (10.3)	41 (26.5)	54 (34.8)	39 (25.2)	0.002
	Female	24 (5.6)	53 (12.3)	164 (38.0)	123 (28.5)	68 (15.7)	
Marital status	Single	10 (6.0)	12 (7.1)	38 (22.6)	63 (37.5)	45 (26.8)	<.001
	Married	19 (4.5)	57 (13.6)	167 (39.9)	114 (27.2)	62 (14.8)	
Education	Illiterate	11 (11.6)	24 (25.3)	51 (53.7)	8 (8.4)	1 (1.1)	<.001
	Primary	5 (5.5)	18 (19.8)	42 (46.2)	16 (17.6)	10 (11.0)	
	Preparatory	3 (3.4)	11 (12.5)	27 (30.7)	34 (38.6)	13 (14.8)	
	Secondary	6 (3.9)	12 (7.9)	44 (28.9)	45 (29.6)	45 (29.6)	
	University or above	4 (2.5)	4 (2.5)	41 (25.5)	74 (46.0)	38 (23.6)	
Dental prosthesis	Yes	13 (6.0)	33 (15.3)	90 (41.7)	54 (25.0)	26 (12.0)	<.001
	No	16 (4.3)	36 (9.7)	115 (31.0)	123 (33.2)	81 (21.8)	
Smoking (N = 310)	Yes	0 (0.0)	5 (13.2)	17 (44.7)	11 (28.9)	5 (13.2)	0.904
	No	9 (3.1)	37 (12.7)	110 (37.7)	88 (30.1)	48 (16.4)	
Qat chewing (N = 310)	Yes	0 (0.0)	15 (12.0)	54 (43.2)	32 (25.6)	24 (19.2)	0.250
	No	9 (4.4)	27 (13.2)	73 (35.6)	67 (32.7)	29 (14.1)	

Chi-square test was used; PGH: perceived general health

Table 4 crosstabulates the PGH against POH. The lower levels of PGH (poor and fair) reported by the participants were significantly associated with lower levels of POH. The vice versa applies to the higher levels of PGH and POH (very good and excellent; *P* < 0.001). The discrepancy of distribution was related to the “good” response. The Spearman’s correlation coefficient was moderate but statistically significant (*r* = 0.486; *P* < 0.001).

**Table 4:** Correlation between responses to perceived oral and general health (POH and PGH) questions (*N* = 587)

		POH					P (χ^2^)	Spearman’s correlation
		Poor	Fair	Good	Very good	Excellent		
PGH	Poor	20 (69.0)	5 (17.2)	2 (6.9)	1 (3.4)	1 (3.4)	<.001	0.486, P < 0.001
	Fair	28 (40.6)	25 (36.2)	13 (18.8)	3 (4.3)	0 (0.0)		
	Good	61 (29.8)	68 (33.2)	64 (31.2)	11 (5.4)	1 (0.5)		
	Very good	17 (9.6)	39 (22.0)	71 (40.1)	44 (24.9)	6 (3.4)		
	Excellent	12 (11.2)	13 (12.1)	25 (23.4)	34 (31.8)	23 (21.5)		

Results of the multiple ordinal regression analysis, as shown in Table 5, revealed better POH among young participants who aged ≤20 and 21–30 years old (*OR* = 3.27, CI_95%_ = 1.16–9.21 and 3.1, CI_95%_ = 1.34–7.19; *P* = 0.025 and 0.008, respectively) compared to those who aged > 50 years old. Participants who were singles showed twice chance of better POH compared to married participants (*OR* = 2.00, CI_95%_ = 1.15–3.48; *P* = 0.014). Contrarily, the participants with lower levels of education (illiterate, primary and preparatory) revealed worse POH (OR = 0.22, 0.32 and 0.42; *P* < 0.001, = 0.001 and 0.01, respectively) compared to the participants who had university level or above.

**Table 5:** Ordinal regression for the effect of study variables on perceived oral health (POH) status

		Estimate	Std. Error	OR	95% CI		Sig.
					Lower	Upper	
Age	≤ 20 years	1.18	0.53	3.27	1.16	9.21	0.025
	21–30 years	1.13	0.43	3.10	1.34	7.19	0.008
	31–40 years	0.79	0.43	2.20	0.94	5.12	0.068
	41–50 years	0.85	0.47	2.34	0.92	5.95	0.073
	> 50 years	Reference					
Gender	Male	0.31	0.28	1.37	0.79	2.36	0.263
	Female	Reference					
Marital status	Single	0.69	0.28	2.00	1.15	3.48	0.014
	Married	Reference					
Education	Illiterate	−1.51	0.42	0.22	0.10	0.50	< .001
	Primary	−1.14	0.33	0.32	0.17	0.61	0.001
	Preparatory	−0.87	0.33	0.42	0.22	0.81	0.010
	Secondary	− 0.18	0.29	0.84	0.47	1.49	0.551
	University or above	Reference					
Dental prosthesis	Yes	−0.34	0.22	0.71	0.46	1.10	0.127
	No	Reference					
Qat chewing	Yes	−0.30	0.25	0.74	0.46	1.21	0.230
	No	Reference					

Pseudo R-Square: Nagelkerke = 0.26; Model Fitting Information: χ2 = 92.45, P < 0.001

In context of PGH, the multiple regression analysis, as shown in Table 6, revealed somewhat different results. Males reported better PGH compared to females (*OR* = 1.61, CI_95%_ = 1.10–2.35; *P* = 0.015). However, education stayed a significant determinant of PGH; the participants with lower levels of education (illiterate and primary) showed worse PGH (OR = 0.15 and 0.28, respectively, *P* < 0.001) compared to those who had university level or above.

**Table 6:** Ordinal regression for the effect of study variables on perceived general health (PGH) status

		Estimate	Std. Error	OR	95% CI		Sig.
					Lower	Upper	
Age	≤ 20 years	0.31	0.40	1.36	0.63	2.97	0.436
	21–30 years	0.36	0.33	1.43	0.76	2.72	0.269
	31–40 years	0.23	0.33	1.25	0.66	2.38	0.488
	41–50 years	−0.14	0.36	0.87	0.42	1.77	0.691
	> 50 years	Reference					
Gender	Male	0.47	0.19	1.61	1.10	2.35	0.015
	Female	Reference					
Marital status	Single	0.22	0.20	1.25	0.84	1.86	0.272
	Married	Reference					
Education	Illiterate	−1.89	0.29	0.15	0.08	0.27	<.001
	Primary	−1.28	0.26	0.28	0.17	0.46	<.001
	Preparatory	−0.49	0.26	0.61	0.37	1.01	0.057
	Secondary	− 0.16	0.21	0.85	0.56	1.29	0.447
	University or above	Reference					
Dental prosthesis	Yes	−0.25	0.17	0.78	0.56	1.08	0.127
	No	Reference					

Pseudo R-Square: Nagelkerke = 0.21; Model Fitting Information: χ2 = 128.79, P < 0.001

## Discussion

To the best of our knowledge, this is the first study among Yemeni people that explored the relationship between POH and PGH, and the relation of each of them with some sociodemographic variables and habits including smoking and Qat chewing. Overall, the correlation between POH and PGH was evident, similarly as were the relationships between these two measures and the other sociodemographic variables. Despite being not significant in the multiple ordinal regression analyses, higher proportions of Qat chewers and smokers rated their POH as poor and fair in comparison to their counterparts. This was not true with regard to PGH - Qat chewing and smoking habits didn’t appear to be significant determinant of PGH.

In the current study, up to half of the participants (51%) rated their POH as good, very good or excellent. This proportion is lower than that reported by other populations: Saudia Arabia (75.1%) [28], South Africa (76.3%) [29], Brazil (74.3%) [30], Australia (83%) [31], and Nigeria (58.3%) [33]. Poor or fair POH were reported by 49% of participants. This proportion could be considered to be high and might be attributed to many factors like age, knowledge and practice of oral health, or cost of dental services. Moreover, this perception might be indifferent unless associated with pain or disfigurement. Contrariwise, only 16.7% of the study population rated their PGH as fair or poor – up to 83% rated it as good, very good and excellent. The former result is lower than that reported by Okanseri et al. among Somalian individuals (38%) [34]. The diversity of results could be explained by the differences in participants’ ages, health knowledge and awareness, and quality of health services among the different populations. Moreover, the lack of healthcare programs, low economic status, and poor nutrition among Yemeni population may reflect negatively on their responses. There might be a high tolerance to systemic diseases in a way that make Yemenis indifferent. Such a tolerance is not applied to oral diseases probably owing to the associated disability. Furthermore, the subjective rating has a chance of bias owing to individuals’ variability in rating themselves [35].

In agreement with many previous studies [36, 37], younger Yemeni participants (21–30 years old) rated their oral and general health as being better than that of elders. This might be related to the fact that elder individuals become unable to maintain good oral hygiene. In addition, in comparison to young adults, elderly experience many age related health problems along with the physiologic changes of body organs that progress with age and decrease the ability of the body to tolerate and resist disease processes. For its part, the education was found to be an important determinant of perceived health status- both PGH and POH. Individuals with higher education levels rated their oral health as being better than that of illiterate and low educational levels. It is well-known that educated persons have higher levels of knowledge, awareness, attitude and beliefs of health issues, have no difficulties in reading and/or understanding healthcare instructions, and they can easily get more facilities and resources to learn more. However, this result is in contrast with that of Kotha et al., who did not find association between education and POH [28].

In contrast to many previous studies [38, 39], our results revealed better PGH and POH among singles in comparison to the married individuals. However, our results were similar to that of Roherer et al. [38] who revealed better self-rated health among unmarried women in comparison to the married ones. On the other hand, Zeng and Thomas [39] in their study found that married individuals tend to overestimate their health status. This may be attributed to the fact that single subjects tend to care more and to look better. Typically, married individuals have more responsibilities and more important concerns that being given priorities over their health status. In context of relation with gender, more females than males reported poor oral and general health. Although this is the case in studies conducted by Szwarcwald et al. and Darviri et al. [36, 37], a possible explanation for this result might be that females usually pay more concern to their general health and oral appearance which may be reflected on their rating.

The bivariate analysis revealed a borderline significant difference (*P* = 0.051) in favor of non-smokers compared to smokers in reporting their oral health status. Such a finding, to some extent, confirms the results of many previous studies, suggesting a direct association between smoking and the prevalence and severity of mucosal, dental, and periodontal disease [40, 41]. However, upon adjusting with the multivariate analyses, the role of smoking was no longer evident. Otherwise, there was a significant difference in favor of Qat non-chewers compared to Qat chewers in relation to their oral health status. Although still controversial, there has been an increasing evidence on the negative effects of Qat on oral and dental conditions including white lesions, mucosal pigmentation, periodontal diseases, tooth loss, plasma cell stomatitis, and xerostomia [42, 43]. More specifically, in context of our study, Qat has been found to be associated with worse self-rated health, lower quality of life, and negative effect on the individual’s general health [44–49]. The latter is not applied to our study where no significant difference was found between Qat chewers and non-chewers in reporting their general health status.

Multiple regression analyses confirmed the roles of age, gender, marital status, and education in determining the self-rated oral and general health. That is, older individuals, females, and lower educated individuals rated their health to be worse than that of their counterparts. Szwarcwal et al. [50] in their national health survey in Brazil, and Darviri et al. [37] in their study reported similar roles for the age, education and gender on perception of health status. Such results were also observed in many previous studies [51–53]. In contrast, Darker et al. [54] did not reported on effects of age, gender, and marital status on self-perception of health. As a rule, disparities in socioeconomic variables definitely lead to wide varieties in health perception [55–58]. As expected, the correlation between POH and PGH was significant; the better the PGH, the better the POH, and vice versa. So long as it is said that each is a mirror of the other. Oral health has been described as “the window to general health”. In many instances, oral cavity may be the early site, and it reflects many signs, of many general systemic diseases like general infections, and nutritional deficiencies [59, 60]. Garcia et al. [61] reported that the greater the number of missing teeth the poorer the quality of life. In line with that, poor dentation leads to difficulty in mastication which ultimately results in poor nutritional intake. Although patients perceive their health statuses from a somewhat different point of view of that of the clinicians, their subjective assessment is of utmost importance and must not be ignored; rather it must be incorporated with the objective measures of disease. In addition, dental patients’ report of their oral health status is a part of their overall reporting of general health status, although still within narrower limits when the general health is, let’s say, reported by medical patients. That is, medical patients will concern more about their general health and treatment [62]. Aside from the measured determinants in this study, the differences in results can be also attributed to many not-included determinants such as: health knowledge and awareness, socioeconomic status and the quality of health services among the different populations. In this context, the lack of healthcare programs, low economic status, and poor nutrition among Yemeni population may reflect negatively on their responses.

Although the study found a significant relationship between POH and PGH and also significant relations with some risk factors, it has some potential limitations. First, the external validity -generalizing the results to all Yemeni dental population- is not fully guaranteed owing to the fact that recruiting consecutive patients doesn’t represent the population of interest. Second, no clinical examination was performed which can further support the relationship between subjective and objective measures. Third, the effect of the other sociodemographic factors, i.e. income and occupation, were not explored in this study. It is well known that Yemen is a poor developing country with a very low Human Development Index of 0.452 (ranked 178th) which entails substantial impact on Yemeni population health, and on health services. This implies, even partially, that most of the included participants might be from low income class, and such an inference must be considered cautiously when presenting our results. Lastly, the cross-sectional nature of the study which makes it difficult to explore the cause-effect relationship. Such limitations suggest further longitudinal follow-up and/or case-control studies which could be helpful in this matter.

Finally, the current situation that the Yemeni population are facing must be emphasized. Yemen goes through the world’s largest humanitarian crisis that undoubtedly has a great effect on the individuals’ perceptions of their oral and general health status. The International Committee of the Red Cross (ICRC) had a substantial concern about the high numbers of people needing treatment and about ongoing outbreaks of diseases such as cholera, diphtheria, and meningitis owing to the current war there [63].

## Conclusion

Within the limitations of the current study, the following conclusions can be drawn:

- Higher levels of oral health problems can be anticipated among patients who perceive poor general health, and vice versa.
- The age, marital status and education were independent determinants of POH, while the gender and education were independent determinants of PGH.
- To some extent, neither Qat chewing nor smoking habits appear to be relevant factors determining the perceived oral and general health status of the Yemeni dental patients.

## Data Availability

The datasets supporting the findings of this article are available from the corresponding author.

## Abbreviations

GH: General Health
ICRC: The International Committee of the Red Cross
OH: Oral Health
OHIP: Oral Health Impact Profile
OHRQoL: Oral Health-Related Quality of Life
PGH: Perceived General Health
POH: Perceived Oral Health
QoL: Quality of Life
